# Quantitative analysis of 3-Tesla magnetic resonance imaging in the differential diagnosis of breast lesions

**DOI:** 10.3892/etm.2014.2154

**Published:** 2014-12-22

**Authors:** ZHEN-SHEN MA, DA-WEI WANG, XIU-BIN SUN, HAO SHI, TAO PANG, GUI-QING DONG, CHENG-QI ZHANG

**Affiliations:** 1 Department of Medical Imaging, Qianfoshan Hospital Affiliated to Shandong University, Jinan, Shandong 250014, P.R. China; 2 Department of Epidemiology and Health Statistics, School of Public Health, Shandong University, Jinan, Shandong 250012, P.R. China

**Keywords:** breast lesions, dynamic contrast-enhanced magnetic resonance imaging, vascular function parameters, pathological grading, differential diagnosis

## Abstract

The aim of this study was to investigate the value of quantitative 3-Tesla (3T) magnetic resonance (MR) assessment in the diagnosis of breast lesions. A total of 44 patients with breast lesions were selected. All the patients underwent MR plain scanning and T1 dynamic contrast-enhanced imaging. The vascular function parameters of the lesions, namely volume transfer constant (Ktrans), rate constant (Kep), extravascular extracellular volume fraction (Ve) and integrated area under the curve (iAUC), were acquired. These parameters were compared between benign and malignant breast lesions, and also among differential grades of invasive ductal carcinoma. The values of Ktrans, Kep and iAUC were significantly different between the benign and malignant tumors; however, the values of Ve in the benign and malignant tumors were not significantly different. The values of Ktrans, Kep and iAUC in invasive ductal carcinoma were significantly different between grade I and grade II, and between grade I and grade III; however, there was no significant difference between grade II and grade III. The Ve values in invasive ductal carcinoma did not significantly differ among grades I, II and III. Among the vascular function parameters, Ktrans exhibited the highest sensitivity and specificity in the differentiation of benign and malignant lesions. Quantitative 3-T MR assessment is valuable in the diagnosis of benign and malignant breast lesions. It can also provide reference values for the differentiation of the histological grade of breast invasive ductal carcinoma.

## Introduction

Breast cancer is one of the most common malignant diseases endangering women’s health. The prevalence of breast cancer has continued to rise in recent years, and the individuals affected have been getting progressively younger. Breast magnetic resonance imaging (MRI) has high sensitivity and specificity (1,2). In the 1970s, Folkman (3), a professor from Harvard University, proposed that tumor growth and metastasis depended on angiogenesis, and since then, numerous in-depth studies of the blood supply to tumors have been conducted using MRI (4–13). Studies using dynamic contrast-enhanced magnetic resonance imaging (DCE-MRI) to investigate breast lesions have been carried out mostly on the qualitative or semi-quantitative analysis level, and those providing a quantitative analysis are relatively few. Quantitative analysis has been performed to evaluate the vascular endothelial permeability and increase in blood flow of tumor tissues for diagnosis, for differential diagnosis and for the evaluation of neoadjuvant chemotherapy for malignant tumors (14,15). This involves analyzing the volume transfer constant (Ktrans), rate constant (Kep), extravascular extracellular volume fraction (Ve), integrated area under the curve (iAUC) and other vascular function parameters, which are of particular interest in the quantitative analysis of DCE-MRI.

In the present study, 3-Tesla (3T) DCE-MRI was used to scan benign and malignant breast lesions, and the differences in vascular function parameters between benign and malignant breast lesions and among various pathological grades of invasive ductal carcinoma quantitatively analyze were quantitatively analyzed.

## Materials and methods

### Clinical data

A total of 44 cases admitted to Qianfoshan Hospital of Shandong University between September 2012 and April 2013 were selected. The breast lesions of the cases were diagnosed by physical examination, ultrasound or mammography X-ray detection as well as by breast DCE-MRI examination. All patients were female, aged from 23 to 68 years old with mean age of 41 years. The patients included in the study had no contraindications to MRI, such as implanted magnetic devices. Patients who had received any neoadjuvant chemotherapy or who had renal failure were excluded. All patients who underwent MR examination were asked to provide signed informed consent prior to the inspection. Finally, 44 patients (total of 44 lesions) were pathologically confirmed by surgery or biopsy, including 30 cases of malignant tumors (68.2%) comprising 26 cases of invasive ductal carcinoma (5 cases of grade I, 14 cases of grade II and 7 cases of grade III), 2 cases of inflammatory breast cancer, 1 case of invasive lipid-rich carcinoma and 1 case of high level ductal carcinoma. The remaining 14 patients had benign tumors (31.8%), including 5 cases of fibroadenoma, 3 cases of breast hyperplasia with a fibroadenoma formation tendency, 2 cases of breast hyperplasia, 1 case of intraductal papilloma, 1 case of mammary hyperplasia with severe inflammation in certain areas, 1 case of borderline phyllodes tumor and 1 case of lipoma. This study was conducted in accordance with the Declaration of Helsinki. This study was conducted with approval from the Ethics Committee of Shandong University (Jinan, China). Written informed consent was obtained from all participants.

### Equipment and parameters

All patients underwent breast MR scanning and T1 dynamic contrast-enhanced MRI. A Siemens Magnetom Skyra 3T MR scanner (Siemens, Erlangen, Germany) and a dedicated eight-channel phased bilateral breast coil were used. The patients were prone, and their bilateral breasts were naturally draped within the coil.

### Conventional scanning

Following conventional horizontal, sagittal and coronal positioning and scanning, horizontal position T1 fat-suppression sequences [repetition time (TR)/echo time (TE), 6.0 msec/2.62 msec; field of view (FOV), 360 mm; matrix, 448×448; slice thickness, 1.2 mm; layer space, 0.24 mm; stimulation number, 1; flip angle, 20°], horizontal position T2-Dixon sequences (TR/TE 7,210 msec/102 msec; FOV, 340 mm; matrix, 320×320; slice thickness, 4 mm; layer space, 0.8 mm; stimulation number, 1; flip angle, 120°) and horizontal diffusion bit sequences (TR/TE, 4,300 msec/63 msec; FOV, 340 mm; matrix, 220×220; slice thickness, 5 mm; layer space, 1 mm; stimulation number, 4 times) were performed.

### T1 dynamic contrast-enhanced scanning

Original T1 scanning (T1 mapping) was initially performed, followed by an enhanced T1 line sequential scan. T1 map scanning was performed by multi-flip angle technology with the following parameters: TR/TE, 7.84 msec/3.37 msec; FOV, 340 mm; matrix, 224×224; slice thickness, 1.5 mm; layer space, 0.3 mm; stimulation number, 1; flip angle, 3°/16°. The continuous T1-enhanced sequence scanning parameters were as follows: TR/TE, 5.61 msec/1.74 msec; FOV, 340 mm; matrix, 224×224; slice thickness, 1.5 mm; layer space, 0.3 mm; stimulation number, 1; flip angle, 10°; total scan time, 22 times; single phase scan time, 30.1 sec with total time of 11.15 min. Following the second phase data collection, 20 ml gadolinium diethylenetriaminepentacetate (DTPA; Haibo Lecco Xinyi Pharmaceutical Co., Ltd., Shanghai, China) contrast agent was intravenously injected at high pressure, followed by a 20-ml injection of saline at a flow rate of 5 ml/sec. Then, 20 phases were continuously collected.

### Data processing and analysis

The scanned images were transmitted to Siemens workstation SYGNO VE40A; post-processing work was performed using TISSUE 4D software. The most evident breast artery or internal thoracic artery was selected to obtain the arterial input function (AIF). The vascular function parameters of the lesions were measured by selecting a region of interest (ROI), as shown on the pseudo-color images.

### ROI selection

The substantive component of a mass was selected as the region of interest, and three levels were selected for each lesion (the level of the maximum cross-sectional area, and one at each of the upper and lower levels), avoiding necrotic tissue, voids, vascular calcification and other features. For each vascular permeability parameter, the average of the parameter at the three levels was taken as the vascular permeability parameter of the lesion.

### Statistical analysis

SPSS software version 7.0 (SPSS, Inc., Chicago, IL, USA) was used to conduct the statistical analysis. Data are expressed as mean ± standard deviation. The vascular function parameters between benign and malignant tumors were compared using Student’s t-test or adjusted t-test. The vascular function parameters among different pathological grades of invasive ductal breast cancers were compared using repeated measures analysis of variance. P<0.05 was considered to indicate a statistically significant difference.

## Results

### Differences in Ktrans, Kep and iAUC between benign and malignant breast lesions

**Figure 1:**
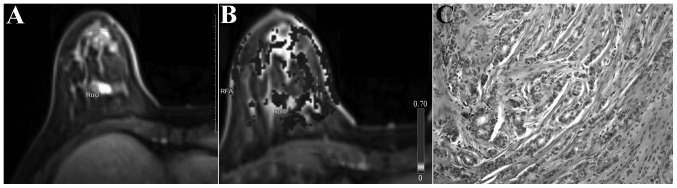
Invasive ductal carcinoma of grade I in the right breast. (A) Early dynamic contrast-enhanced image, (B) image of vascular function parameters and (C) pathological image (hematoxylin and eosin stained; magnification, ×200).

**Figure 2:**
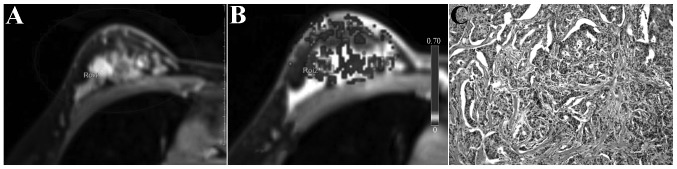
Invasive ductal carcinoma of grade II in the right breast. (A) Early dynamic contrast-enhanced image, (B) image of vascular function parameters and (C) pathological image (hematoxylin and eosin stained; magnification, ×200).

**Figure 3:**
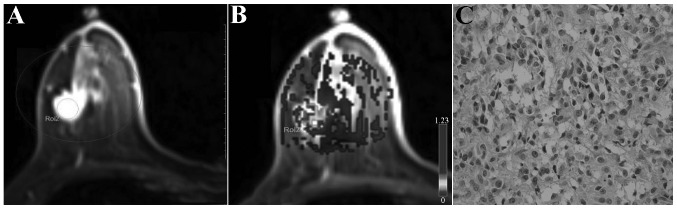
Invasive ductal carcinoma of grade III in the right breast. (A) Early dynamic contrast-enhanced image, (B) image of vascular function parameters and (C) pathological image (hematoxylin and eosin stained; magnification, ×200).

**Figure 4:**
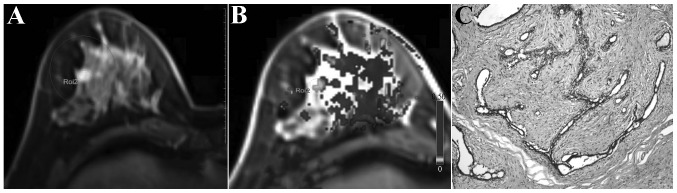
Fibroadenoma in the right breast. (A) Late dynamic contrast-enhanced image, (B) image of vascular function parameters and (C) pathological image (hematoxylin and eosin stained; magnification, ×200).

The Ktrans, Kep and iAUC values of the malignant breast lesions were significantly higher than those of the benign lesions, and had slight differences between the different levels of invasive ductal carcinoma. Malignant breast lesions generally were significantly enhanced in the early enhancement stage, and red (representing higher perfusion) was observed in lesion regions of the dynamic contrast-enhanced pseudo-color maps; the higher the value of perfusion parameters, the larger the relative scope of the red zone and the deeper the color. In addition, the higher the relative value of the high perfusion parameters, the deeper the invasive ductal carcinoma malignancy degree (represented in black and white in Figs. 1–3). The benign breast lesions generally exhibited delayed enhancement, and yellow (representing lower perfusion) was observed in lesion regions of the dynamic contrast-enhanced pseudo-color maps (represented in black and white in Fig. 4).

### Significance of vascular function parameters in the differential diagnosis of benign and malignant breast lesions

The differences in Ktrans, Kep and iAUC between benign and malignant tumors were statistically significant (P<0.05). The difference in Ve between benign and malignant tumors was not statistically significant (P>0.05, Table I).

### Significance of vascular function parameters in the grading of breast invasive ductal carcinoma

The differences in Ktrans, Kep and iAUC between grade I and grade II, and between grade I and grade III of ductal carcinoma were statistically significant (P<0.05). The differences in Ktrans and Kep between grade II and grade III of invasive ductal carcinoma were not statistically significant (P>0.05). No statistically significant differences in Ve were observed among grades I, II and III of invasive ductal carcinoma (P>0.05, Table II).

### Diagnostic value of vascular function parameters at 95% confidence

The lower bounds at 95% confidence of the Ktrans, Kep and iAUC values in malignant cancers were defined as the boundary values for the respective differential diagnosis of benign and malignant lesions. Ktrans, Kep and iAUC had high sensitivity and specificity in the differential diagnosis of benign and malignant lesions, of which Ktrans had the superior diagnostic performance with the highest sensitivity and specificity (Table III).

## Discussion

Perfusion-weighted imaging (PWI) is an imaging technique that reflects tissue or microvascular lesion distribution and blood flow perfusion, which is able to evaluate angiogenesis in tumor tissues *in vivo* in a non-destructive manner and is extremely valuable for studying tumor blood supply (16). DCE-MRI is a PWI technology that can indirectly reflect the functional status of tumor blood vessels, thereby providing a reliable basis for further diagnosis and treatment by revealing the microvascular perfusion status and the degree of tissue vascularization. The pathological basis of the perfusion effect includes changes in the number of tumor vascular vessels, and changes in blood vessel function. Malignant tumors can release vascular endothelial cell growth factor to induce capillary growth. For such tumors, it has been observed that neovessel density is high, an early tumor perfusion effect is evident, neovascular walls are incomplete, and contrast agent rapidly bleeds into the extracellular space to result in rapid filling of the tumor with contrast agent and a fast outflow rate (17). However, for benign tumors, vascular provision is relatively less, and the contrast agent infusion effect is not evident (18).

DCE-MRI can dynamically display the pharmacokinetic changes of contrast agent in the vessels as a continuously obtained series of images. Currently, the blood dual-chamber kinetic model (dual-chamber representation of the microvascular and extravascular interstitium) proposed by Tofts *et al* (19) is widely used to perform DCE-MRI. The results are analyzed to obtain the following vascular function parameters: Ktrans, Kep, Ve and iAUC. iAUC is actually a semi-quantitative parameter that is associated with blood flow into the tumor, tumor perfusion and tumor tissue spaces (20,21), and can effectively reflect changes in Ktrans, Kep and Ve.

Ocak *et al* (22) demonstrated that the Ktrans and Kep values of prostate malignancy were significantly higher than those of benign lesions. Yao *et al* (23) identified that Ktrans and Kep differed significantly between normal bowel walls and rectal cancer. In addition, the authors observed statistically significant differences in Ktrans between colorectal cancer with lymph node metastasis and that without lymph node metastasis, and among different Dukes stage of colorectal cancer, while Kep and Dukes’ stage had a moderate correlation. Li *et al* (24) found that Kep was able to predict the efficacy of the first cycle of neoadjuvant chemotherapy in breast cancer patients. Nilsen *et al* (25) observed that Ktrans and Kep had a statistically significant difference between breast cancer lesions associated with bone metastasis and that without bone metastases; the perfusion and vascular permeability of breast cancer lesions with metastasis were significantly lower than those of non-metastatic breast cancer lesions. The present study demonstrated that the Ktrans, Kep and iAUC values of malignant breast lesions were significantly higher than those of benign lesions; this may be associated with the biological characteristics of the lesions. The malignant lesions were vigorously growing with increased tumor angiogenesis, increased microvessel density and structural disorder, partially incomplete neovascular endothelium and abnormal vascular endothelial cell morphology. They also exhibited enlarged spaces between the endothelium and basement membrane, basement membrane and vascular pericytes. Thus, the angiogenesis of malignant lesions was associated with a high permeability (26). For benign lesions, the neovascularization was less than that of malignant lesions, and vascular endothelium growth was relatively complete. Therefore, for malignant lesions, the diffusion rate of contrast agent from intravascular to extravascular tissues and the diffusion rate of contrast agent from extravascular tissues back to vessels were significantly greater than those of benign lesions, which was reflected as higher Ktrans and Kep values for malignant lesions than for benign lesions.

Tofts (27) found that the Ve value was less consistent than the other vascular function parameters; this was considered to be due with the fact that Ve is often affected by edema around the lesion. Certain scholars also consider that this may be associated with the slow rate of change in the relative proportions of extravascular extracellular volume within the organization in the process of disease development, resulting in the Ve value range between benign and malignant lesions having a degree of overlap (28,29). The present study demonstrated that Ve values did not significantly differ between benign and malignant lesions, which is associated the characteristics of Ve, as it represents the percentage of contrast agent retained in tissue spaces. Ktrans, Kep and Ve satisfy the following relationship: Ve = Ktrans/Kep. The neovascular permeability of malignant lesions is high, resulting in increased Ktrans and Kep values; however, the increase in the proportions of the two values is uncertain. Therefore, the Ve values of benign and malignant lesions did not exhibit a significant difference.

The study further analyzed the vascular function parameters among different pathological grades in invasive ductal carcinoma. It was found that the differences in Ktrans and Kep among grades I, II and III of invasive ductal carcinoma were statistically significant, while no statistically significant differences of Ktrans and Kep were observed between grades II and III of invasive ductal carcinoma. The Ve values demonstrated no statistically significant differences among grades I, II and III of invasive ductal carcinoma. Tumor formation and development can be divided into two stages, namely the clonal proliferation stage of tumor cells and the tumor angiogenesis-promoting sustainable growth phase (30). Breast cancer is pathologically graded through duct/gland formation, nuclear pleomorphism and mitotic count. Grade I invasive ductal carcinoma comprises mostly duct/gland formation and early mitotic stage. At this grade, the endothelial integrity is relatively good. The vascular endothelial differentiation of grade II and III invasive ductal carcinoma is poor with high permeability and increased perfusion resistance; however, as grades II and III are characterized by high permeability, there may be some overlap in the monitoring parameters of tumor blood vessel growth of the two grades. Furthermore, the number of cases was few, which may lead to the generation of errors.

Baek *et al* (31) found that Ktrans had relatively greater significance among the vascular function parameters for the differential diagnosis of benign and malignant lesions. The present study found that the diagnostic sensitivity was 80.0% when Ktrans was 0.832 min^–1^, and the specificity was 92.9%, both of which were higher than the values for Kep and iAUC. Therefore, Ktrans was more meaningful in differentiating benign and malignant lesions, and can provide a quantitative indicator for clinical diagnosis.

In conclusion, this study compared vascular function parameters between benign and malignant breast lesions and among different levels of invasive ductal carcinoma and found that vascular function parameters (Ktrans, Kep and iAUC) were meaningful in the differential diagnosis of benign and malignant breast lesions. The sensitivity and specificity of Ktrans were the highest, and had a certain degree of significance in the grading of invasive ductal carcinoma. However, this study included a relatively small number of patients; a larger sample should be investigated in future studies. The optimal imaging parameters of dynamic contrast-enhanced MR imaging of the breast were collected and analyzed to obtain optimal vascular function parameters. Thus, the diagnosis and differential diagnosis of benign and malignant breast lesions was investigated.

## Figures and Tables

**Table I:** Comparison of vascular function parameters between benign and malignant groups.

Group	n	Ktrans (min^−1^)	Kep (min^−1^)	Ve	iAUC
Benign	14	0.136±0.088	0.291±0.185	0.565±0.196	4.633±3.687
Malignant	30	0.967±0.361^a^	1.742±0.552^a^	0.581±0.137	14.614±5.692^a^

Values shown are mean standard ± deviation.

a P<0.05 vs. the benign group.

Ktrans, volume transfer constant; Kep, rate constant; Ve, extravascular extracellular volume fraction; iAUC, integrated area under the curve.

**Table II:** Comparison of vascular function parameters among different histological grades of invasive ductal carcinoma.

Grading	n	Ktrans (min^−1^)	Kep (min^−1^)	Ve	iAUC
Grade I	5	0.579±0.030	1.199±0.050	0.480±0.016	7.737±0.170
Grade II	14	1.196±0.268^a^	2.000±0.503^a^	0.628±0.118	14.558±4.054^a^
Grade III	7	1.011±0.242^a^	2.001±0.312^a^	0.556±0.199	20.465±1.936^a^

Values shown are mean standard ± deviation.

a P<0.05 vs. grade I.

Ktrans, volume transfer constant; Kep, rate constant; Ve, extravascular extracellular volume fraction; iAUC, integrated area under the curve.

**Table III:** Efficacy of Ktrans, Kep and iAUC in the diagnosis of benign and malignant lesions.

Parameters (%)	Boundary value	Sensitivity (%)	Specificity (%)
Ktrans (min^−1^)	0.832	80.0	92.9
Kep (min^−1^)	1.536	66.7	86.7
iAUC	12.488	73.3	85.7

Ktrans, volume transfer constant; Kep, rate constant; iAUC, integrated area under the curve.
